# Aspects cliniques thérapeutiques et pronostiques des tumeurs germinales malignes de l’ovaire: expérience tunisienne de 21 ans

**DOI:** 10.11604/pamj.2020.36.178.23916

**Published:** 2020-07-13

**Authors:** Imen Ghaddab, Raja Briki, Sassi Bouguizene, Hedi Khairi

**Affiliations:** 1 Service de Gynécologie et d´Obstétrique, Hôpital Universitaire Farhat Hached, Sousse, Tunisie

**Keywords:** Ovaire, tumeurs germinales malignes, chirurgie, chimiothérapie, survie, pronostic, fertilité, Ovary, malignant germ cell tumors, surgery, chemotherapy, survival, prognosis, fertility

## Abstract

**Introduction:**

les tumeurs germinales malignes de l´ovaire (TGMO) sont des tumeurs ovariennes rares. Chaque type histologique peut avoir des particularités cliniques et thérapeutiques qu´il est important de connaître. L´objectif était de rapporter et analyser les particularités des données épidémiologiques, diagnostiques, anatomopathologiques, thérapeutiques et pronostiques des TGMO dans notre contexte tunisien.

**Méthodes:**

notre étude est une enquête rétrospective descriptive et analytique réalisée aux services de Gynécologie-obstétrique du CHU FARHAT HACHED de Sousse sur une période de 21 ans colligeant tous les cas des patientes ayant été traitées pour TGMO.

**Résultats:**

un nombre total de 30 dossiers a été éligible pour notre étude. L´âge moyen de nos patientes était de 22ans. La majorité des patientes étaient en période d´activité génitale. Le motif de consultation était représenté essentiellement par les douleurs abdomino-pelviennes suivie d´une augmentation du volume abdominal. L´échographie abdominopelvienne a été pratiquée chez 80% de nos patientes montrant un aspect suspect de malignité chez 100% d´entre elles. Pour nos patientes, 70% ont été abordées par laparotomie médiane vu le volume tumoral et 30% seulement par cœlioscopie. 76,7% ont eu un traitement conservateur. On a noté la prédominance de stade I alors qu´on n´a pas eu de cas en stade IV. La survie globale tout stade confondu était de 96,7% à 2ans et de 85,7% à 5ans et 75,8% à 10ans. Les facteurs pronostiques des TGMO isolés de notre série étaient le délai de consultation supérieur à 6mois, l´âge supérieur à 30ans, la taille tumorale supérieure à 20cm et le stade tumoral.

**Conclusion:**

il serait plus intéressant de rassembler les autres cas de TGMO diagnostiqués au niveau des autres registres du cancer du pays afin d´établir un registre national des tumeurs rares de l´ovaire.

## Abstract (en)

**Introduction:**

malignant germ cell tumors of the ovary (TGMO) are rare ovarian tumors. Each histological type can have clinical and therapeutic particularities which it is important to know. The objective was to report and analyze the particularities of the epidemiological, diagnostic, anatomopathological, therapeutic and prognostic data of TGMO in Tunisian context.

**Methods:**

this study is a retrospective descriptive and analytical survey carried out at the Gynecology-Obstetrics departments of the CHU FARHAT HACHED in Sousse over a period of 21years collecting all the cases of patients having been treated for TGMO.

**Results:**

a total number of 30 files was eligible for our study. The average age of our patients was 22years. The majority of patients were in a period of genital activity. The reason for the consultation was mainly represented by abdomino-pelvic pain followed by an increase in abdominal volume. Abdominopelvic ultrasound was performed in 80% of our patients showing a suspicious appearance of malignancy in 100% of them. For our patients, 70% were approached by median laparotomy given the tumor volume and only 30% by laparoscopy. 76.7% had conservative treatment. The predominance of stage I was noted while there was no case in stage IV. The overall survival for all stages was 96.7% at 2years and 85.7% at 5years and 75.8% at 10years. The prognostic factors of the TGMOs isolated from our series were the consultation time greater than 6 months, the age greater than 30years, the tumor size greater than 20cm and the tumor stage.

**Conclusion:**

it would be more interesting to bring together the other cases of TGMO diagnosed in other cancer registries in the country in order to establish a national register of rare ovarian tumors.

## Introduction

Les tumeurs germinales malignes sont des tumeurs embryonnaires qui dérivent de la transformation des cellules germinales primitives. Elles peuvent être gonadiques (ovariennes et testiculaires) dans 66% des cas ou extra gonadiques dans les 34% restant (Sacro coccygiennes, vaginales, rétropéritonéales, médiastinales et cérébrales) [1]. Les tumeurs germinales de l´ovaire sont des tumeurs ovariennes excessivement rares et ne représentent que 2% à 3% des tumeurs malignes ovariennes [1]. De point de vue histologique, les tumeurs germinales malignes de l´ovaire (TGMO) sont composées de plusieurs types tumoraux et sont divisées en deux groupes: les tumeurs germinales malignes séminomateuses appelées aussi les dysgerminomes et les tumeurs germinales malignes non dysgerminomateuses (TGMND) qui sont histologiquement définies par la présence d´au moins un des contingents suivants: la tumeur du sac vitellin, le choriocarcinome, le carcinome embryonnaire et le tératome immature. Les TGMO se différencient, sur le plan diagnostique thérapeutique et pronostique, des adénocarcinomes par de nombreux points: un age de survenue beaucoup plus précoce, puisqu´il s´agit de tumeurs de la fillette et de la jeune femme [2] et il les distingue également par un diagnostic à un stade plus précoce, un meilleur pronostic, avec un taux de survie à 5ans de 100% pour les TGMO séminomateuses et de 85% pour les TGMO non séminomateuses [3] Ces tumeurs se caractérisent aussi par une très grande chimio-sensibilité avec des modalités thérapeutiques particulières, avec une chirurgie le plus souvent conservatrice et des protocoles de chimiothérapie adaptés [4]. Il y a très peu d´études tunisiennes ayant analysé les particularités diagnostiques de ce type de tumeur ainsi que les modalités thérapeutiques et évolutives; alors que la rareté et les difficultés de gestion justifient une étude centralisée sur la stratégie de prise en charge des patientes présentant une TGMO. Pour cela, nous avons mené cette étude, et nous nous sommes fixés les objectifs suivants: rapporter et analyser les particularités des données épidémiologiques, diagnostiques, anatomopathologiques, thérapeutiques et pronostiques des tumeurs germinales malignes de l´ovaire illustrées et traitées dans le service de Gynécologie-Obstétrique du centre hospitalo-universitaire Farhat Hached à Sousse, Tunisie.

## Méthodes

Notre étude est une enquête rétrospective descriptive et analytique réalisée aux services de Gynécologie-Obstétrique, oncologie médicale et anatomo-pathologie du CHU FARHAT HACHED de Sousse sur une période de 21ans du 1^er^septembre 1998 au 30 septembre 2019. L´étude a inclue toutes les patientes qui ont été prises en charge pour TGMO, confirmées histologiquement. Un nombre total de 30 dossiers a été éligible pour notre étude. Les patientes ayant une tumeur germinale maligne de l´ovaire prouvée histologiquement ont été incluses. Les différents types histologiques qui ont été inclus sont le dysgerminome, la tumeur du sac vitellin, le choriocarcinome, le carcinome embryonnaire et le tératome immature, la tumeur germinale mixte. Par contre, les dossiers non exploitables et les autres types histologiques, comme le tératome mature cancérisé, ont été exclus. La méthode de collecte des données a été faite par l´exploitation des dossiers médicaux, les comptes rendu opératoires et d´anatomopathologie des patientes et des comptes rendu du traitement adjuvant (chimiothérapie) transcrites sur une fiche informatisée préétablie. Les données recueillies étaient de nature quantitative et qualitative et les variables explicatives étudiées étaient principalement: Les données épidémiologiques: âge, parité; les antécédents carcinologiques; Les données cliniques: le délai de consultation, les circonstances de découverte, la taille tumorale, les données anatomo-pathologiques; les données thérapeutiques: chirurgie, chimiothérapie et les données évolutives. On a effectué une analyse statistique descriptive des principales variables étudiées sociodémographiques, cliniques et thérapeutiques: les données qualitatives seront exprimées en nombre et en pourcentage, les données quantitatives en moyenne et en écart type. L’ensemble de ces données étaient saisies et traitées à l´aide du logiciel SPSS version 24.0. La survie a été calculée selon la méthode de Kaplan-Meir. Une recherche bibliographique a été réalisée à l´aide des moteurs de recherche Pubmed/Medline, Science Direct, Google Schoor, Google Books et Cochrane datasse. Cette recherche a utilisé essentiellement les mots clés suivants: « ovary », « malignant germ cell tumors, « management », « conservative surgery », « radical surgery », «chemotherapy», « staging » « prognosis » et « fertility ».

## Résultats

**Profil épidémiologique et étude clinique:** trente cas de TGMO ont été recensés pendant la période d´étude. Durant cette même période, on a colligé dans notre service de Gynécologie et Obstétrique au CHU Farhat Hached 717 cas de tumeurs malignes de l´ovaire, c´est qui fait que la fréquence de TGMO a été de 4,1%. La moyenne d´âge des patientes était de 22ans avec des extrêmes allant de 10 à 40ans. Cette moyenne était variable en fonction de type histologique (Tableau 1). Concernant le statut gynéco-obstétrical, 2 de nos patientes, âgées de 10 et 12ans, étaient en préménarche, aucune patiente n´était ménopausée et la majorité des patientes étaient en activité génitale (28 cas) dont 8 étaient mariées et une parmi eux était suivie pour une infertilité primaire de 2 ans. Les moyennes de gestité et de parité chez ces patientes étaient de 1,6 et de 1,2 respectivement. Le motif de consultation était représenté essentiellement par les douleurs abdomino-pelviennes dans 46% des cas et la symptomatologie abdominale aigue était observée chez une patiente avec comme étiologie, la torsion d´annexe. Les autres signes cliniques sont illustrés dans le Tableau 1.

**Tableau 1:** tableau illustrant les caractéristiques cliniques des patientes ainsi que les caractéristiques tumorales radiologiques et histologiques

La moyenne d´âge (ans)		22 [10-40]
Dysgerminomes		17
Tumeurs non dygerminomateuses		25
Antécédents	Antécédents familiaux ou personnels de cancers de l’ovaire	0
	Kystectomie ovarienne droite pour un tératome mature	1
Motif de consultation	Douleurs abdomino-pelviennes	46%
	Augmentation du volume abdominal	17%
	Palpation d'une masse abdominale	12%
	Altération de l´état général	11%
	Trouble du transit	8%
	Troubles urinaires	4%
	Métrorragie	2%
Aspects radiologiques	Aspect solido-kystique	16 (66,7%)
	Aspect hétérogène	21 (87,5%)
	Paroi propre épaissie	13 (54,1%)
	Aspect échogène	9 (37,5%)
	Taille tumorale (mm)	140 [60-280]
	Ascite	9 (37,5%)
	Nodules péritonéaux suspects	2 (8,3%)
	Hyper-vascularisation avec prise du doppler	18 (75%)
Types histologiques tumorales	Dysgerminome	7 (23%)
	Teratome immature	14 (46.7%)
	Carcinome embryonnaire	5 (16,7%)
	Tumeur du sac vitellin	3 (10%)
	Tumeur mixte	1 (3.3%)
Grade histologique pour les tératomes	Grade 1	4 (57%)
	Grade 2	2 (28%)
	Grade 3	1 (14%)

**Explorations radiologiques:** l´échographie préopératoire était réalisée chez 24 patientes (80%) et elle avait montré une masse pelvienne unilatérale dans 87% des cas et dans 2 cas la localisation de la tumeur n´a pu être précisée vue le volume tumoral. Les caractéristiques radiologiques sont résumées dans le Tableau 1. La tomodensitométrie (TDM) thoraco-abdomino-pelvienne n´a été pratiqué que chez 11 patientes dont en 2 cas il y avait un doute de l´origine exacte de la tumeur et dans les autres cas la taille tumorale dépassait 150 mm. Dans le cadre du bilan d´extension, la TDM TAP n´avait pas montré des localisations tumorales péritonéales, hépatiques ou pulmonaires d´allure secondaire ni encore d´atteinte ganglionnaire dans tous les cas.

**Caractéristiques histologiques tumorales** (Tableau 1): sur le plan histologique, la taille tumorale moyenne était de 150mm. La tumeur a été bilatérale dans 2 cas dont un cas était un dysgerminome et le deuxième était un tératome immature. Les patientes étaient classées selon la classification de la FIGO comme suit: La tumeur était classée stade I et III dans respectivement 56,7 et 36,7% des cas. Le stade II n´a représenté que 6,6% des cas. Aucune tumeur n´était d´emblée métastatique. La répartition des différents types histologiques selon la classification de la FIGO est représentée dans le (Tableau 2).

**Tableau 2:** répartition des types histologiques selon la classification de la FIGO

		Stade								
		lA	IB	IC	IIA	IIB		IIIA	IIIB	IIIC
Carcinome embryonnaire	Effectif	0	0	0	0	0	0		1	4
	%	0,0%	0,0%	0,0%	0,0%	0,0%	0,0%		20%	80%
Dysgerminome	Effectif	1	1	2	1	0	0		0	2
	%	14,3%	14,3%	28,6%	14,3%	0,0%	0,0%		0,0%	28,6%
Tératome immature	Effectif	8	1	3	0	0	1		0	1
	%	57,1%	7,1%	21,4%	0,0%	0,0%	7,1%		0,0%	7,1%
Tumeur du sac vitellin	Effectif	0	0	1	0	1	0		0	1
	%	0,0%	0,0%	33,3%	0,0%	33,3%	0,0%		0,0%	33,3%
Tumeur germinale mixte	Effectif	0	0	0	0	0	0		1	0
	%	0,0%	0,0%	0,0%	0,0%	0,0%	0,0%		100%	0,0%
Total	Effectif	9	2	6	1	1		1	2	8
	%	30%	6,7%	20%	3,3%	3,3%		3,3%	6,7%	26,7%

**Traitement chirurgical:** pour le stade I, le traitement chirurgical initial était conservateur laissant en place un ovaire et l´utérus chez 16 patientes (94,1%), et radical d´emblée chez une patientes (59%). Pour le stade II, 100% des patientes ont eu un traitement conservateur. La biopsie de l´ovaire controlatérale ainsi que les biopsies péritonéales n´ont pas été réalisées dans tous les cas de stade I et II. Les gestes effectués pour les tumeurs stade III étaient une biopsie ovarienne dans 63,3% des cas, une annexectomie dans 36,3% et des biopsies péritonéales dans 90,9% des cas. La biopsie ovarienne était bilatérale dans 71,4%. Le traitement chirurgical était radical chez 07 patientes (23,3%) et conservateur laissant en place un ovaire et l´utérus chez 23 patientes (76,7%) (Tableau 3). Dans le groupe de patientes ayant une TGMO découverte à un stade précoce, une reprise chirurgicale a été réalisée pour une stadification et pour totalisation chirurgicale chez 3 patientes ayant eu déjà leur capital d´enfants. Parmi les patientes ayant une TGMO découverte à un stade avancé, une chirurgie radicale a été réalisée dans 3 cas: une d´entre elles a eu une chimiothérapie néoadjuvante pour une tumeur germinale mixte stade IIIC avec une réponse incomplète (réduction tumorale estimée à 50% avec marqueurs tumoraux négatifs); la chirurgie était complète sans résidu tumoral mais le curage ganglionnaire pelvien et lomboaortique était positif; les deux autres étaient des dysgerminomes stade IIIC ayant une chimiothérapie néoadjuvante avec une réponse complète pour une et partielle pour l´autre. La chirurgie n´était pas complète pour cette dernière. Le curage ganglionnaire a été réalisé chez ces deux patientes et était positif chez une. Tous ces actes étaient réalisés par voie laparotomique.

**Tableau 3:** type de chirurgie selon les stades de FIGO

		TRAITEMENT	
		Radical	Conservateur
lA	Effectif	1	8
	%	11,1%	88,9%
IB	Effectif	1	1
	%	50%	50%
IC	Effectif	2	4
	%	33,3%	66,7%
IIA	Effectif	0	1
	%	0,0%	100%
IIB	Effectif	0	1
	%	0,0%	100%
IIIA	Effectif	0	1
	%	0,0%	100%
IIIB	Effectif	1	1
	%	50%	50%
IIIC	Effectif	2	6
	%	25%	75%
Total	Effectif	7	23
	%	23,3%	76,7%

**Traitement adjuvent:** parmi les cas consultés, six patientes n´ont pas eu une chimiothérapie adjuvante et une surveillance rigoureuse a été recommandée chez 4. Un traitement complémentaire de l´intervention chirurgicale à type de polychimiothérapie a été indiqué chez 14 patientes (46,6%) alors que 10 patientes (33,3%) ont eu une chimiothérapie néoadjuvante. Une chimiothérapie à base de sels de platine était réalisée dans 100% des cas. Le protocole BEP (Bléomycine- Etoposide- Cisplatine) a été utilisé dans 21 cas, le BVP (Bléomycine- Vinblastine- Cisplatine) chez 2 patientes et VIP (Etoposide + Ifosfamide+ cisPlatine) chez une patiente. Les nombres de cures étaient repartis entre 2 et 5 cures. La chimiothérapie néoadjuvante a été indiquée pour tous les tumeurs classée stade III. La radiothérapie a été réalisée chez une seule fillette impubère, âgée de 10 ans et le champ d´irradiation était les chaine ganglionnaires lombo-aortique.

**Evolution:** dans notre série, il a été noté une poursuite évolutive chez une patiente ayant une résection incomplète post chimiothérapie d´un dysgerminome stade IIIC. Cette patiente avait développé une métastase hépatique, et une chimiothérapie de deuxième ligne a été mise en place. Nous avons observé 3 cas de récidives métastatiques qui sont survenus après un délai moyen de 48 mois. Les deux cas de récidives ont été observés chez des patientes n´ayant pas reçues de traitement adjuvant et elles s´étaient évadées. La troisième récidive était sous forme métastatique d´une tumeur germinale mixte stade IIIB ayant eu une chirurgie radicale avec un curage pelvien et lombo aortique. Le traitement a consisté en une chimiothérapie dans tous les cas.

**Figure 1:**
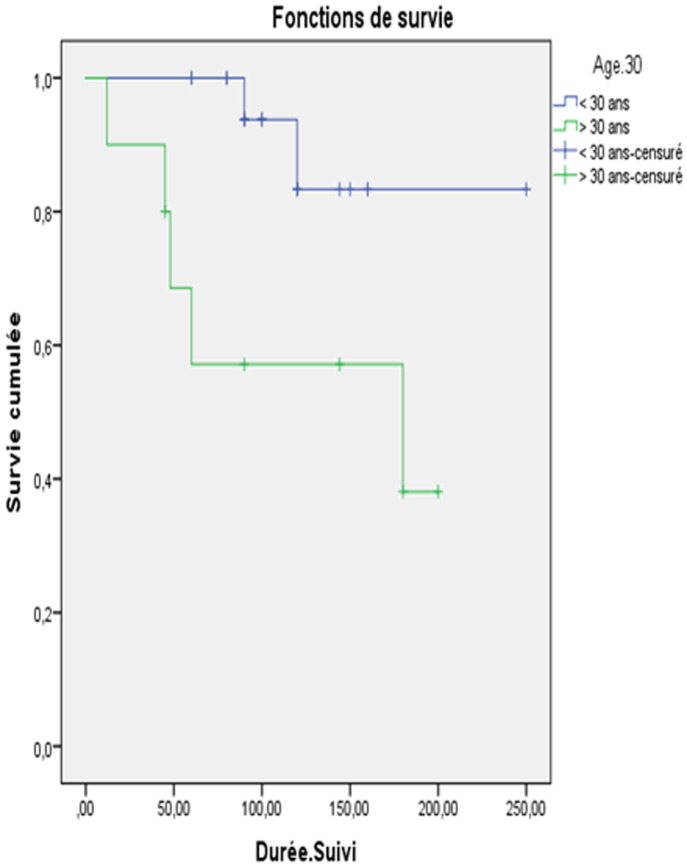
la survie globale en fonction de l´âge

**Figure 2:**
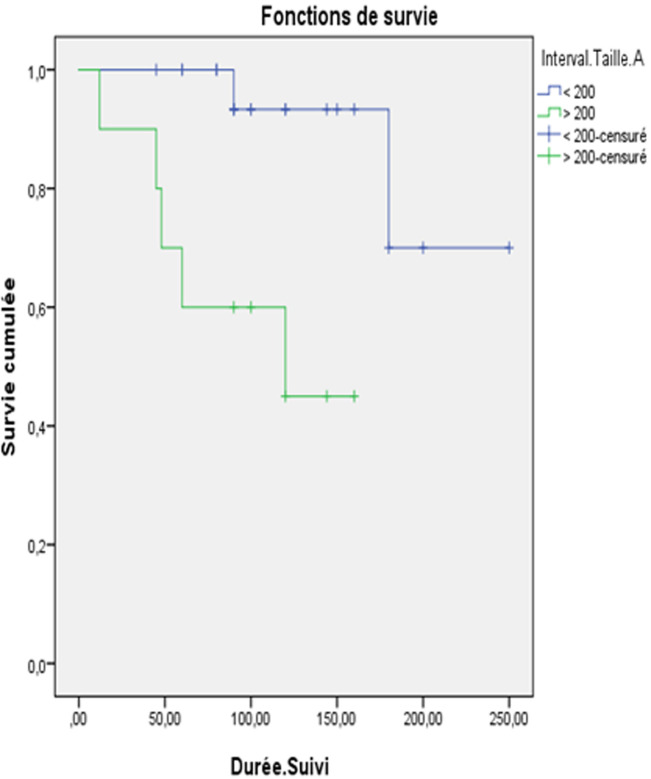
la survie globale en fonction de la taille tumorale

**Survie:** sur les 28 patientes, 21 étaient en rémission complète, soit 75% des cas. On a noté 7 cas de décès ayant survenus entre 12 mois et 180 mois après la chirurgie (Tableau 4). La survie globale tout stade confondu était de 96,7% à 2 ans et de 85,7% à 5ans et 75,8% à 10ans. L´âge était un facteur pronostique important (P = 0,011). La survie globale à 2 et à 5 ans était meilleure chez les patientes âgées de moins de 30ans. Elle était de 100%. Alors que pour les patientes âgées de plus de 30ans, elle était à 85% à 5ans et 68% à 5ans (P = 0,029) (Figure 1). Concernant la taille tumorale, la survie globale pour les patientes ayant une tumeur de taille = 20cm était meilleure à celles ayant des tumeurs de taille > 20cm (P = 0,004) (Figure 2). Pour le stade, la survie globale à 5 et à 10ans des patientes classées stade I était de 94,7% et celle des patientes classées stade II était de 100%. Chez les patientes classées stade III, la survie globale à 5ans était de 73,2% et 62,4% (P = 0,014). A propos le type histologique, la survie globale était meilleure pour les TGMO non dysgerminomateuses par rapport aux TGMO dysgerminomateuses à 2ans et à 10ans. Cette différence n´est pas statistiquement significative (P=0,053). La survie globale était légèrement meilleure en cas de tératome immature parrapport aux autres TGMND (P = 0,054). La survie globale à court terme (2 et 5 ans) était comparable entre les patientes ayant un traitement conservateur et celles ayant un traitement radical. Par contre, elle était, à long terme (10ans), meilleure chez les patientes ayant un traitement conservateur: 83% versus 5%.

**Tableau 4:** le décès en fonction du type histologique, stade et éventualités thérapeutiques

Stade	Type histologique	Traitement chirurgical	Chimiothérapie	Type	Nombre	Décès	Survie (mois)
IA	Tératome immature	conservateur	NON			OUI	60
IIIA	Tératome immature	conservateur	NON			OUI	45
IIIB	Tumeur germinale mixte	radical	Neoadjuvente	VIP	5	OUI	120
IIIC	Carcinome embryonnaire	conservateur	Neoadjuvente	BEP	2	OUI	180
IIIC	Dysgerminome	radical	Neoadjuvente	BEP	4	OUI	90
IIIC	Tumeur du sac vitellin	conservateur	Neoadjuvente	BEP	2	OUI	48
IIIC	Dysgerminome	Radical	Neoadjuvente	BEP	4	OUI	12


BEP: Bloémycine- Etoposide- Cisplatine VIP: Etoposide - Ifosfamide- cisplatine

**Fertilité:** parmi les 17 patientes qui ont déjà survécu et qui ont bénéficié d´un traitement conservateur, 9 ont réussi à avoir une grossesse dont 7 étaient spontanées et 2 induites (pour une infertilité d´origine masculine). Pour les sept autres patientes, 5 sont encore célibataires et non mariées, 2 avaient déjà des enfants et actuellement sont sous dispositif intra-utérin au lévonorgestrel.

## Discussion

Notre étude est une enquête rétrospective descriptive qui a permis de recueillir les différentes données cliniques, histologiques et thérapeutiques d´une pathologie ovarienne assez rare qui est la pathologie germinale maligne de l´ovaire (TGMO). La taille de l´échantillon est de 30 patientes atteintes de TGMO. Il serait intéressant de faire l´étude sur une série plus importante en nombre, mais vu que la pathologie en question est très rare, une étude prospective semble difficile et non faisable. Il s´agit néanmoins de la plus grande série tunisienne se rapportant aux TGMO. Les TGMO sont des néoplasies gynécologiques rares. Elles représentent environ 2% à 3% de tous les cancers de l´ovaire dans les pays occidentaux et 29% de l´ensemble des tumeurs germinales malignes. Elle survient principalement chez les femmes jeunes avec un taux d´incidence de 75% pour les femmes âgées de moins de 30 ans [5]. Le dysgerminome est la variante histologique la plus fréquente des TGMO et elle représente 40% des tumeurs germinales malignes de l´ovaire [6]. Dans notre étude leur fréquence était de 23%. Le registre de surveillance, d´épidémiologie et de résultats finaux (SEER) de l´institut national du cancer, a signalé que le tératome immature était la forme la plus fréquente de TGMND représentant 35,6%, suivi par la tumeur du sac vitellin (14,4%), et moins fréquemment la tumeur à cellules germinales mixtes (5,3%) et le carcinome embryonnaire (4,1%) [7]. Les choriocarcinomes sont exceptionnellement rares et représentent 2,1% à 3,4% de tous les TGMO [7]. Nous n´avons relevé aucun cas de choriocarcinome à l´état pur ni impur dans notre série et au contraire de ce qui avait été précédemment rapporté par plusieurs auteurs, le tératome immature était le type de TGMO le plus fréquemment représenté (46,7%). À propos les circonstances de découverte, de nos jours, il n´existe pas des symptômes et de signes pathognomoniques pour les TGMO et ces signes sont souvent multiples, subtils et non spécifiques. Selon la majorité des auteurs, la douleur abdominale associée à une masse pelvienne-abdominale palpable reste le symptôme majeur et il est présent chez environ 85% des patientes [8]. Les autres signes, moins courants, sont la distension abdominale (35%), la fièvre (10%), l´ascite (10%) et les saignements vaginaux (10%) [9].

Pour les explorations radiologiques, l´échographie pelvienne est l´examen paraclinique clé pour l´exploration des masses annexielles et en particulier des tumeurs ovariennes. Elle oriente vers l´organicité de la tumeur et permet d´étayer une présomption de malignité par l´analyse morphologique de la tumeur ovarienne ainsi que par la mise en évidence d´une ascite, d´adénopathies pelviennes et/ou de métastase hépatiques ou péritonéales. De même, dans notre travail l´échographie abdominopelvienne a permis de montrer un aspect suspect de malignité chez 100% d´entre elles. La tomodensitométrie (TDM) n´est pas toujours nécessaire au diagnostic, c´est un complément non obligatoire de l´échographie dans le diagnostic et l´établissement du bilan d´extension des tumeurs ovariennes. Elle permet de rattacher une masse à développement abdomino-pelvien à l´ovaire. La TDM ne constitue pas le bon moyen d´exploration des petites tumeurs mais il reste indispensable pour le bilan d´extension préopératoire, pour la surveillance postopératoire et pour établir le diagnostic précoce des récidives. Entre autre, le scanner abdominopelvien permet de mieux explorée les aires ganglionnaires. L´imagerie par résonnance magnétique occupe de plus en plus une place dans l´exploration des tumeurs pelviennes. Sa sensibilité tissulaire permet une parfaite délimitation anatomique et elle permet de caractériser plus finement la lésion, et cela est spécialement important lorsqu´il existe une composante dysgerminomateuse [10]. Concernant la prise en charge thérapeutique, l´objectif essentiel du traitement de TGMO est de guérir les patientes tout en préservant la fonction hormonale ovarienne et la fertilité ultérieure en minimisant la toxicité des traitements. Ainsi, la chirurgie occupe une place importante dans le traitement des TGMO dont le but de la chirurgie est triple: diagnostique, stadification et thérapeutique. La procédure chirurgicale est progressivement devenue moins invasive dans le but de préserver les possibilités de grossesses ultérieures, même en cas de stades évolués. Le geste chirurgical consiste donc au minimum en: une annexectomie unilatérale, une exploration complète du pelvis et de toute la cavité abdominale, un lavage péritonéal et/ou un prélèvement de toute ascite présente lors de l´ouverture de l´abdomen, des biopsies péritonéales systématiques (y compris au niveau de l´épiploon) et un prélèvement de tout élément suspect (une biopsie de l´ovaire controlatéral si lésion suspecte et des biopsies des ganglions rétropéritonéaux) [11]. Depuis plus de deux décennies et devant la grande chimio sensibilité de ces types de tumeurs, la chimiothérapie type BEP est le schéma standard à tous les stades de la maladie [9] et le protocole BEP décrit quatre cycles, toutes les trois semaines, avec bléomycine: 30 mg (J1, 8, 15), Etoposide: 100 mg/m^2^/J (J1, 5) et Cisplatine: 20 mg/m^2^/J (J1, 5). Ce protocole a considérablement amélioré le taux de survie: 100% chez les patientes atteintes d´une TGMO à un stade précoce et 75% chez celles atteintes d´une TGMO à un stade avancé [9]. Ceci a été démontré aussi par notre étude. Inversement à la chimiothérapie, la radiothérapie est presque abandonnée de nos jours en raison de ses effets secondaires et de faite que la chimiothérapie soit plus efficaces, beaucoup moins toxique et elle permet la préservation de la fonction ovarienne [12]. Dans notre série, la radiothérapie était réalisée chez une seule patiente âgée de 10 ans ayant un dysgerminome stade IIA. Bien que les récidives des dysgerminomes soient rares, 75% se produiront au cours de la première année suivant le traitement initial [13]. Seulement quelques cas de récidives survenant après les deux premières années ont été rapportés dans la littérature [14] et contrairement aux dysgerminomes, les tumeurs germinales malignes non dysgerminomateuses réapparaissent dans les deux premières années dans 90% des cas. Les TGMND ont un mauvais pronostic lorsqu´elles rechutent, avec un taux de survie à long terme de 10% [13]. Le carcinome embryonnaire et la tumeur du sac vitellin sont les tumeurs les plus agressives parmi les TGMO. Elles métastasent rapidement aussi bien par voie lymphatique que par voie hématogène et envahissent les organes de voisinage ainsi que toute la cavité péritonéale [12].

Les différents taux de survie globale des TGMO rapportés dans la littérature et même dans étude prouvent le bon pronostic de ces tumeurs et leur assez bonne réponse au traitement. Selon la société américaine du cancer, les taux de survie globale à 5ans pour les différents types de TGMO, varient de 69% pour les stades IV et 98% pour les stades I du FIGO [15]. La survie globale et à 5 ans des TGMO varie considérablement selon le sous type. Les dysgerminomes ont un pronostic très favorable. Aux premiers stades, ils ont un taux de survie à 5 ans de 96,9% [12]. Dans notre série, elle était de 100%. Pour le stade III, les dysgerminomes ont une survie à cinq ans de 61% [12]; Pour les tératomes immatures purs, le taux de survie à 5 ans, tous les stades confondus, est de 70 à 80%, et il est de 90 à 95% pour le stade I [13]. Dans notre série les tumeurs vitellines et les tumeurs germinales mixtes étaient les types histologiques s´associant à des taux de survie les plus bas. Ce qui concorde avec les données de l´étude MITO9 [16]. La fertilité est une question centrale compte tenu de l´âge jeune, voire très jeune, du diagnostic chez des patientes en général nullipares et présentant une pathologie d´excellent pronostic global. Tous les auteurs s´accordent actuellement sur la nécessité d´un traitement permettant de préserver la fertilité des jeunes patientes atteintes de TGMO. Globalement, les résultats sur les fonctions hormonales ovariennes et sur la fertilité des patientes ayant été traitées par chirurgie conservatrice et chimiothérapie sont bons. Ainsi, dans les études récentes portant sur les TGOM, environ 75% des femmes ont réussi à concevoir un enfant [17, 18]. Le taux d´infertilité rapporté chez les femmes tentant de concevoir après le traitement pour TGMO varie de 5% à 10% [19]. Ce taux est similaire au taux d´infertilité dans la population normale [20]. Dans notre étude, parmi dix femmes tentant une grossesse, neuf ont pu concevoir, dont deux présentant un stade III au moment du diagnostic, confirmant ainsi en outre que le traitement conservateur doit être préconisé même aux stades avancés. Les données de notre étude confirment que la fonction gonadique normale et la fertilité sont possibles après une chirurgie conservatrice pour les tumeurs malignes des cellules germinales ovariennes, même en cas de chimiothérapie.

## Conclusion

Bien que l´effectif de cette étude soit réduit, les résultats trouvés semblent être conformes aux séries de plus grand effectif. Mais, il serait plus intéressant de rassembler les autres cas de TGMO diagnostiqués au niveau des autres registres du cancer du pays afin d´établir une série nationale de cette tumeur, et ce dans le cadre d´un registre national des tumeurs rares de l´ovaire.

### Etat des connaissances sur le sujet

- Les tumeurs germinales malignes de l´ovaire (TGMO) sont des tumeurs très rares, hétérogènes, elles touchent principalement la femme jeune;
- La conduite à tenir n´est pas randomisée des tumeurs germinales malignes de l´ovaire tumeurs dans les pays africains notamment la Tunisie.

### Contribution de notre étude à la connaissance

- Il s´agit néanmoins de la plus grande série tunisienne se rapportant aux TGMO;
- Réaliser un état de lieu des TGMO dans le centre tunisien;
- Randomiser et centraliser la stratégie de prise en charge des patientes présentant une TGMO en Tunisie.

## Remerciements

Les auteurs tiennent à remercier tous les participants, le personnel du département d´obstétrique et de gynécologie du CHU Farhat Hached de Sousse, Tunisie.
